# Supplementary material for: Fully 3D printed flexible, conformal and multi-directional tactile sensor with integrated biomimetic and auxetic structure
Source: Commun Eng. 2023 Nov 14;2:80. doi: 10.1038/s44172-023-00131-x (PMC10956052; doi:10.1038/s44172-023-00131-x)
Supplement: Supplementary file 2 — Supplementary material [file 44172_2023_131_MOESM2_ESM.docx]

**Supplementary Information**

**Fully 3D Printed** **Flexible, Conformal and Multi-directional Tactile Sensor with Integrated Biomimetic and Auxetic Structure**

**^1^Department of Mechanical, Aerospace and Civil Engineering, School of Engineering, The University of Manchester, Manchester, M13 9PY, UK**

**^2^Department of Engineering Science, University of Oxford, Oxford, OX1 3PJ, UK**

**^3^Key Laboratory of Bionic Engineering, Ministry of Education, Jilin University, China**

**^4^School of Science, Engineering and Environment, University of Salford, Manchester, M5 4BR, UK**

Yuyang Wei-1,2, Bingqian Li-3, Marco Domingos-1, Zhihui Qian-3, Yiming Zhu-1, Lingyun Yan-1, Lei Ren*-1,3 and Guowu Wei*-4

***Correspondence**: [lei.ren@manchester.ac.uk](mailto:lei.ren@manchester.ac.uk) and [g.wei@salford.ac.uk](mailto:g.wei@salford.ac.uk)

**List of Contents**

Experimental procedure of the *in-vivo* and robotic grasping test

Supplementary Figures 1-14

Supplementary Table 1 and 2

Supplementary Movie 1

**Supplementary Methods**

***Experimental procedure of the in-vivo grasping test***

For the *in-vivo* grasping test on the human subject, we selected three extrinsic located in the human forearm and hand that affect hand motion. All three muscle forces were estimated based on electromyography (EMG) signals captured by the Delsys wireless EMG system (Delsys Inc., Boston, US) during the *in-vivo* grasping test. Each Trigno sensor was placed along muscle fibers following the guidelines of surface electromyography for the non-invasive assessment of muscles (See Fig. S11). Before the reactive grasping test, maximum voluntary contraction (MVC) tests were performed for all nine muscles using a Jamar dynamometer. The recorded EMG data were band-pass filtered (20–400 Hz) with a Butterworth filter and rectified. The muscle forces during grasping were then derived based on the maximum voluntary contraction forces and the assumption that for isometric muscle contracting, there is a linear relationship between the EMG signal and muscle force. The subject who participated in this *in-vivo* reactive grasping experiment provided informed consent, which was approved by the Ethics Committee of the First Hospital of Jilin University.


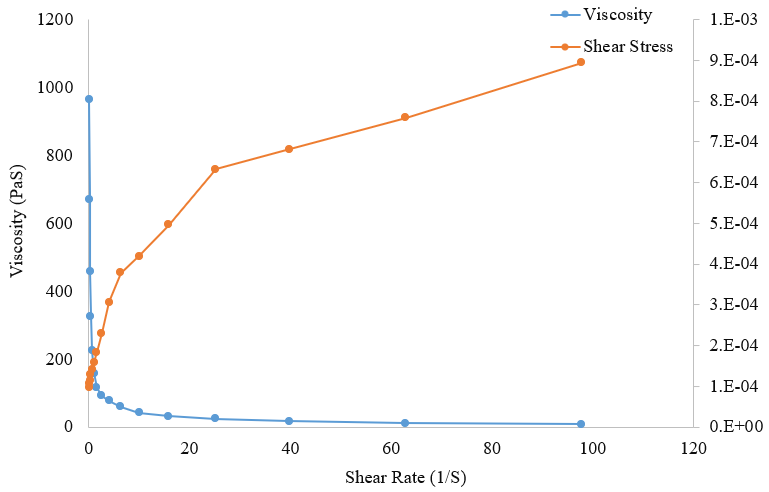


**Supplementary Figure 1 | The viscosity and shear rate of Copper-Silicone composite as a function of shear rate.** The viscosity was decreasing under shear strain (shear thinning) which means this composite could be applied for 3D printing. This amplitude sweep test was carried out by using DHR-30 hybrid rheometer (TA Instruments).


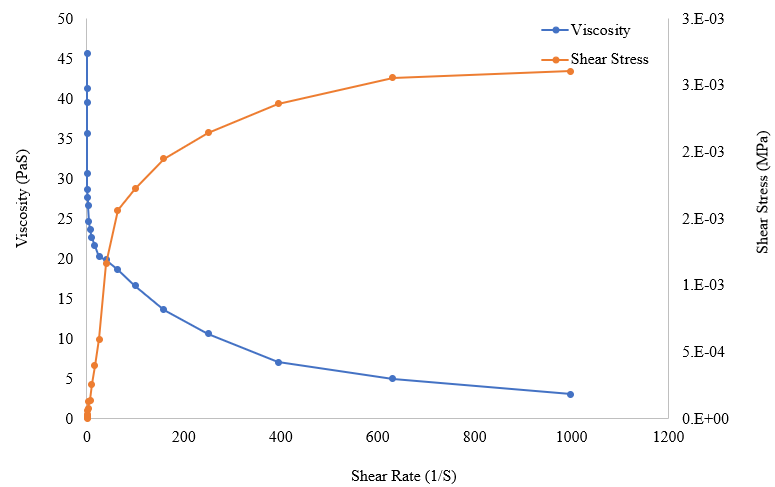


**Supplementary Figure 2 | The viscosity and shear stress of CNT/Graphene/Silicone rubber composite as a function of shear rate.** The viscosity was decreasing under shear strain (shear thinning) which means this composite could be applied for 3D printing.


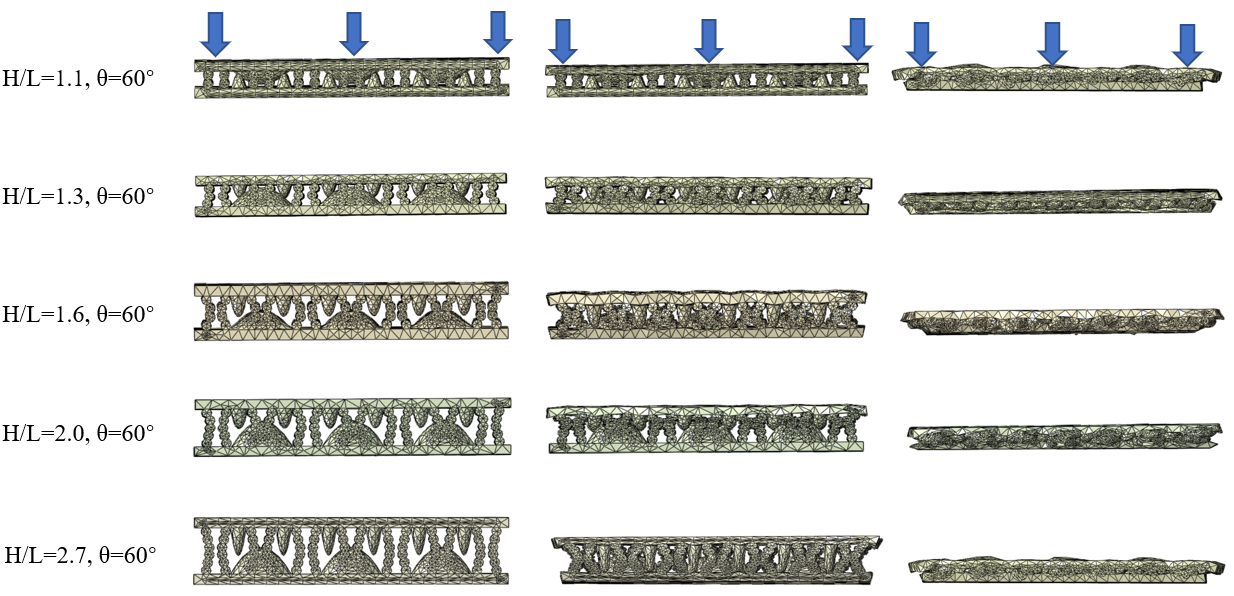
 **Supplementary Figure 3 | The simulation results for optimizing the auxetic structure of the sensor.** The simulation results of the sensors with re-entrant angle of 60^o^ and different H/L ratios are presented. The sensors before, during and after the compression were shown.


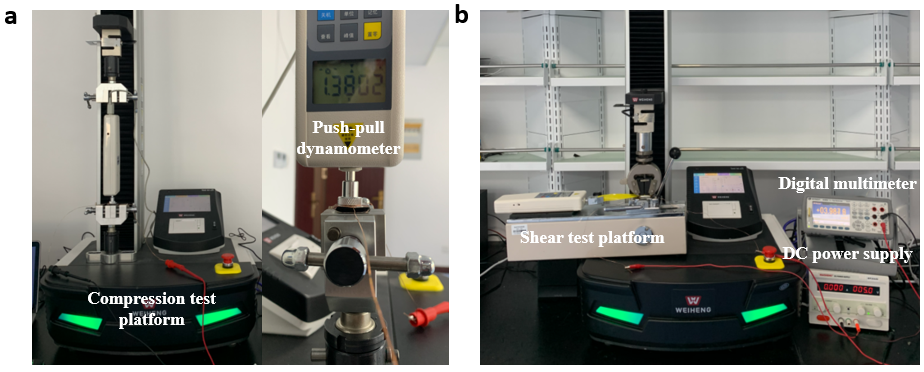


**Supplementary Figure 4 | The testing platform for characterization in terms of the pressure sensor** (**a**) The compression test platform for evaluating normal sensitivity. The universal testing machine and a push-pull dynamometer were used to produce compression force onto the tactile sensor. (**b**) The shear test platform for quantifying shear sensitivity. The horizontal shear test platform with a push-pull dynamometer was mounted on the universal test machine. The push-pull dynamometer produced shear force while the universal testing machine provided normal compression. A DC-power supply was used to produce 5V voltage for the sensor and a digital multimeter was employed to record the current flow over the sensor during the test.


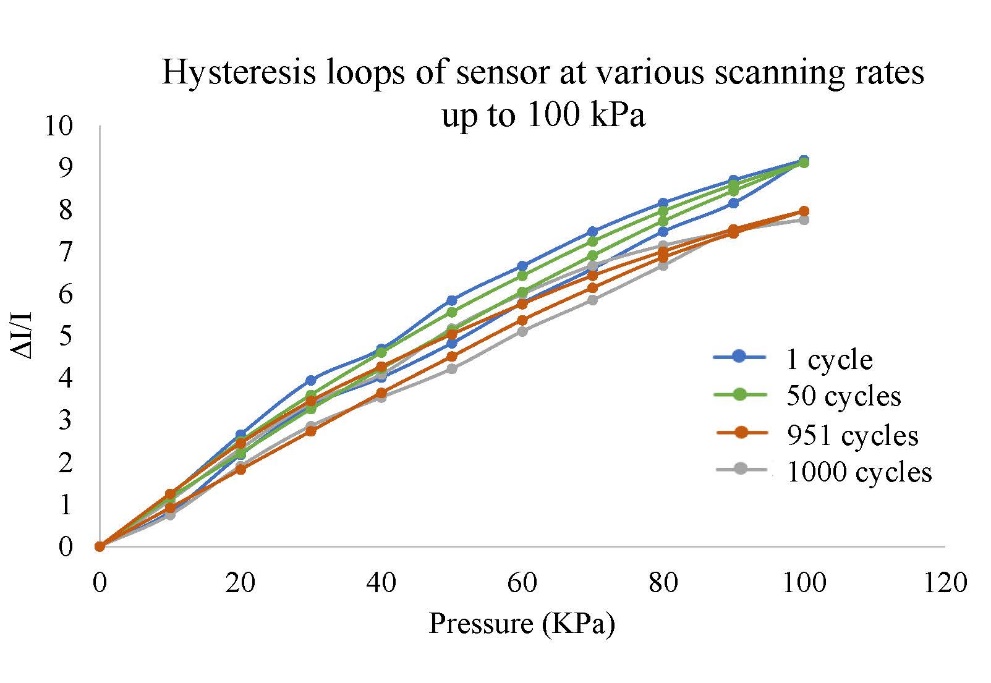


**Supplementary Figure 5 | Hysteresis loops of sensor at various scanning rates up to 100 kPa.**
The hysteresis loops for cycles 1, 50, 951, and 1000 are presented, where the hysteresis was calculated based on the average value of the first and last 50 cycles.


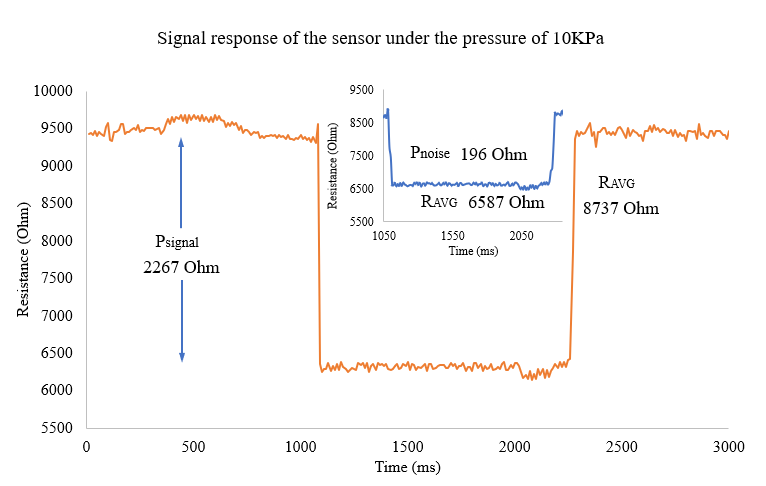


**Supplementary Figure 6 | Signal to noise ratio (SNR) of the sensor under the pressure of 10KPa.** The resistance changes from ∼6500 to ∼8750 Ω, resulting a fractional resistance change ΔR/Ro of ∼0.26. Figure 6b shows a magnified single response pulse, which constitutes the response between 1050 and 2450 ms in Figure 8a. The SNR expressed in dB can be calculated using the equation ${SNR}_{dB}=20log((\Delta R/R_{signal})/(\Delta R/R_{noise}))$, where ΔR/Rsignal is the fractional change in resistance under the pressure of 10KPa and ΔR/Rnoise is the resistance fluctuation under this pressure. The ΔR/Rsignal is calculated to be 2267/8737 = 0.2595, and the ΔR/Rnoise is calculated to be 196/ 6587 = 0.030, therefore, the SNR is 18.7 dB.


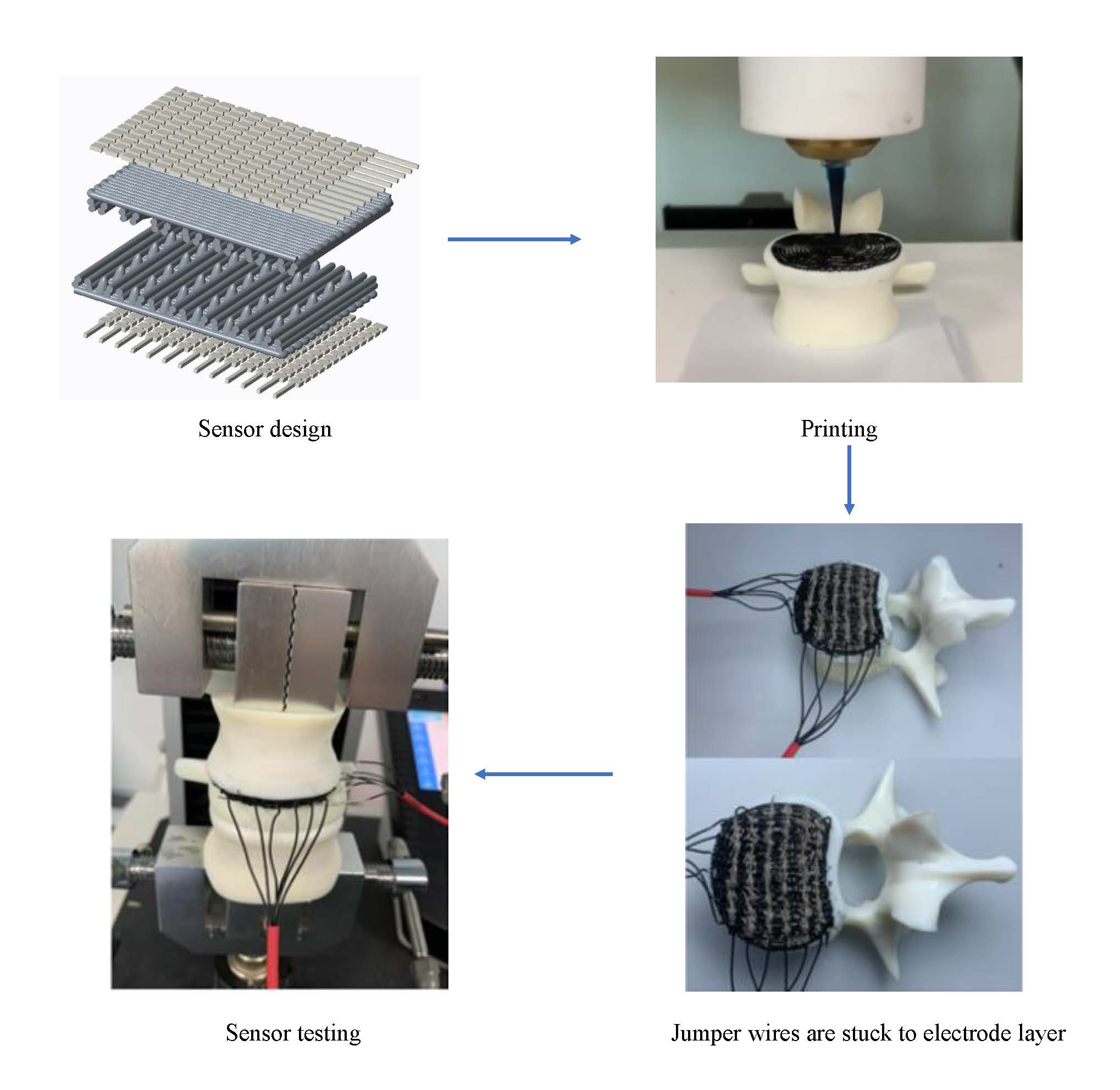


**Supplementary Figure 7 | The general fabrication process of the sensor.** The sensor's structure is first designed and optimized prior to fabrication. The sensor is then printed directly onto the working surface, and the jumper wires are connected to the electrode layer using the same material used for printing the electrode.


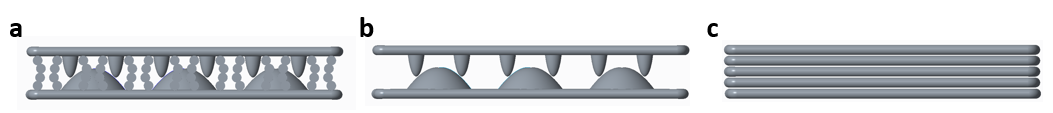


**Supplementary Figure 8 | The three different structures of the sensor.** (**a**) Auxetic and inter-locked structure. (**b**) Inter-locked structure. (**c**) Planar structure.


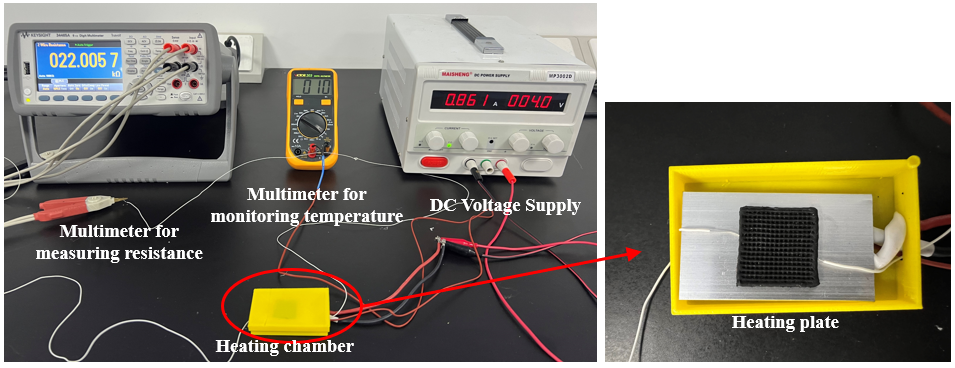


**Supplementary Figure 9 | The testing platform for characterization in terms of the temperature sensor.** A heating chamber consisting of the a plastic box with the dimension of 7.5🞩3🞩cm^3^and a heating plate powered by DC voltage supply was developed to provide a stable temperature variation. The hot plate was powered and heated by a DC voltage supply. The variation of the resistance was recorded by multimeter (Keysight 34465A, Keysight Ltd., HK) at the frequency of 500Hz while the temperature was monitoring by another multimeter.

**Supplementary Figure 10 | The temperature response of the sensor under different pressures.** The sensor was tested under the pressures ranging from 1 to 200KPa. This data shows that temperature variations within the range of 20 to 40℃ have negligible effects on the sensor's performance, as supported by our experimental results.


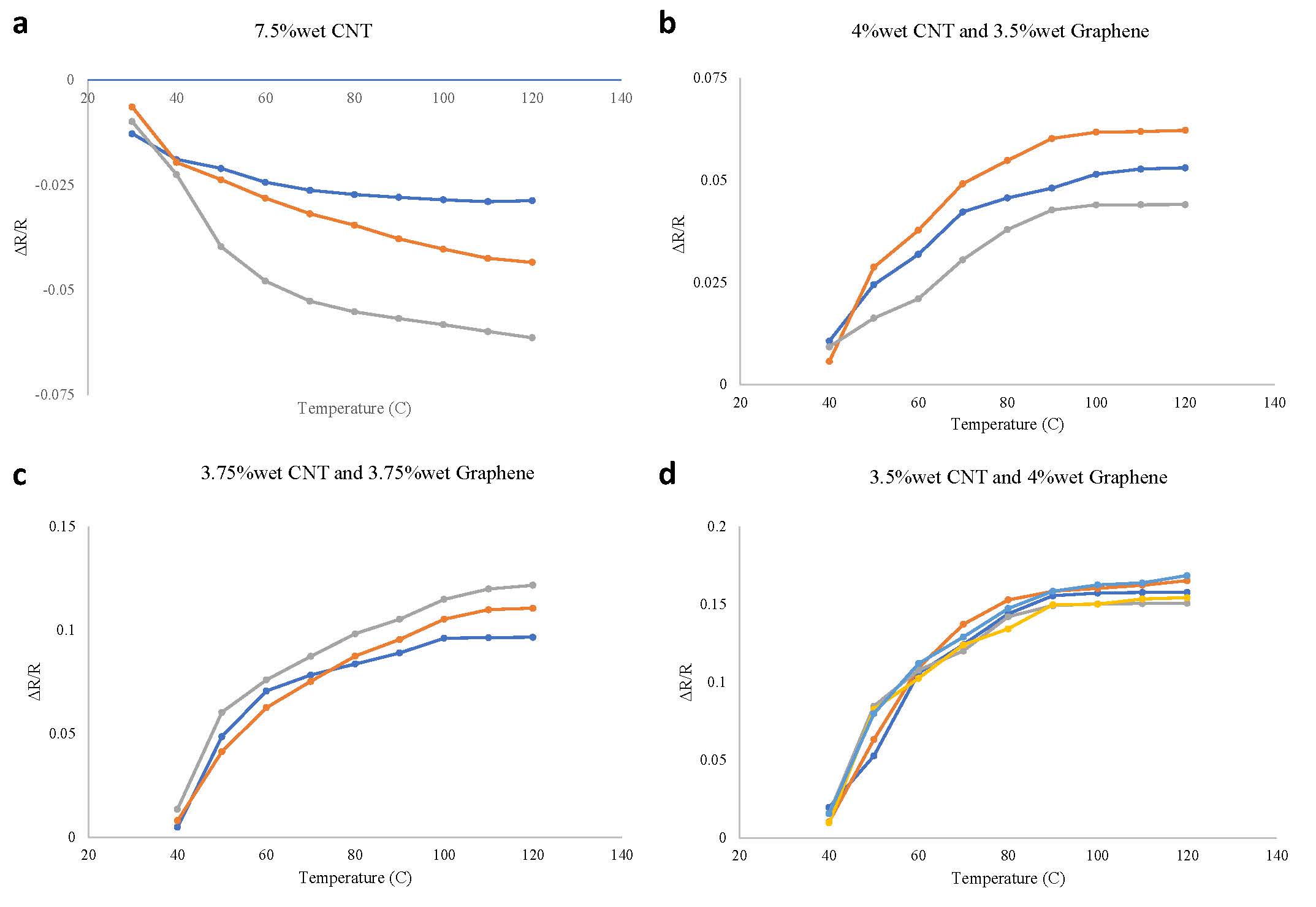


**Supplementary Figure 11 | The optimization of the weight ratio of carbon nano tube to graphene. (a)** The response of the sensor based on the composite material contains 7.5% wt of CNT. Negative temperature coefficient of resistance (TCR) was presented. Unstable signal response were obtained based on three sensor samples. **(b)** The response of the sensor based on the composite material contains 4% wt of CNT and 3.5% wt of graphene. The TCR values varied largely among the three sensor samples. **(c)** The response of the sensor based on the composite material contains 3.75% wt of CNT and 3.75% wt of graphene. The TCR values are still varied largely among the three sensor samples. **(d)** The response of the sensor based on the composite material contains 3.5% wt of CNT and 3.75% wt of graphene. The TCR values were improved while unstable thermoresistive properties were observed based on the responses of three sensor samples.


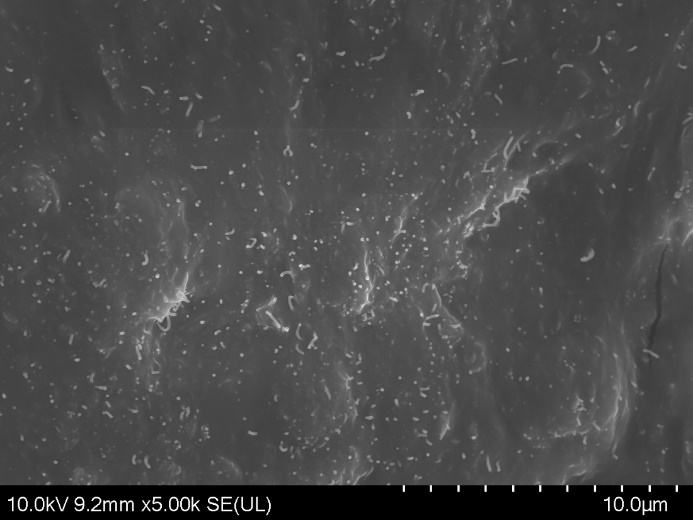


**b**

**a**


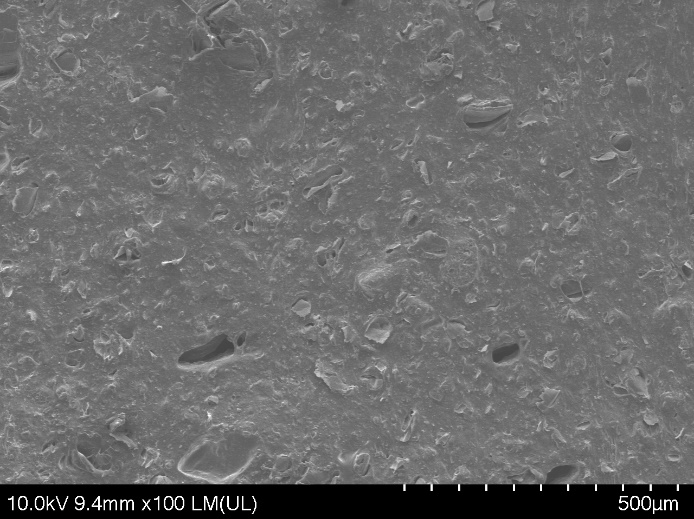


**c**

**c**


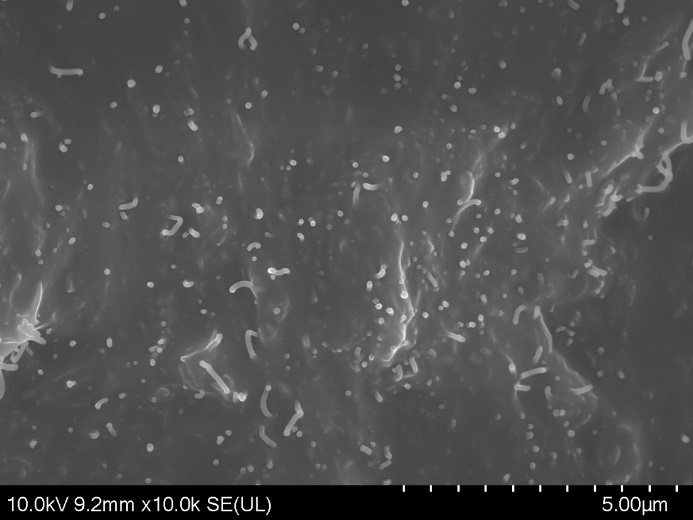

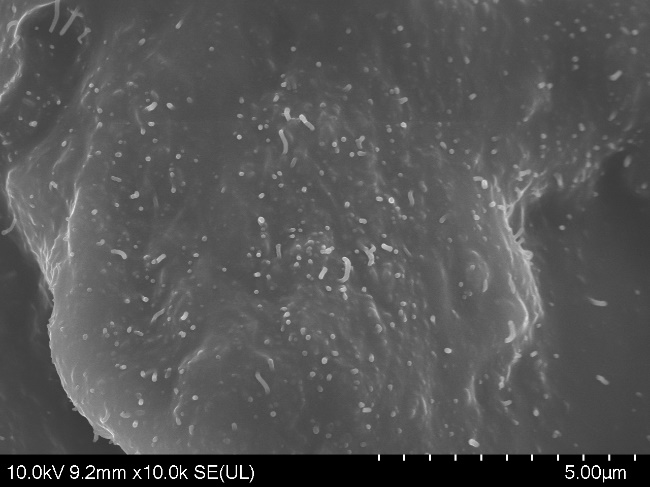


**d**

**Supplementary Figure 12 | The images taken by scanning electron microscope (Hitachi regulus 8220, Japan) . the CNT and graphene palate are evenly distributed in the silicone matrix and no obvious aggregation of CNT was found. (a)** The micrograph taken with 100 times magnification. **(b)** The micrograph taken with 5,000 times magnification **(c). (d)** The micrograph taken with 10,000 times magnification**.**


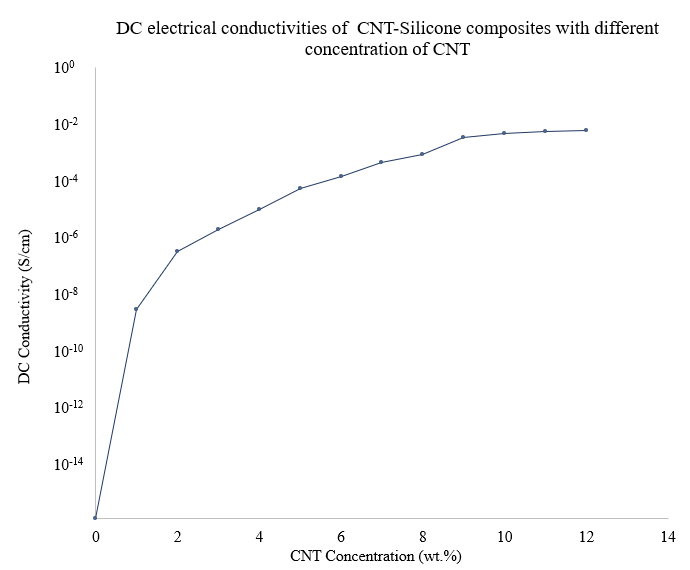


**Supplementary Figure 13 | The electrical conductivities of CNT-Silicone composites under different concentration of CNT.** The weight percentage of CNT was tested till 13%.


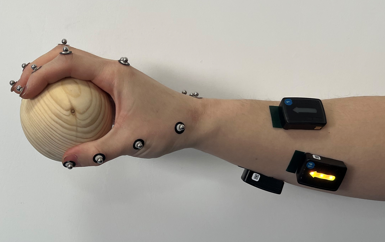


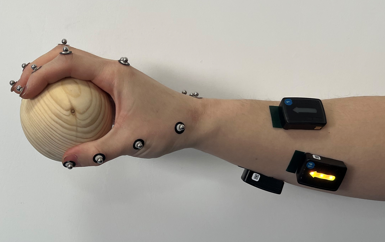

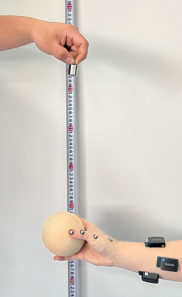


**b**

**a**

**Supplementary Figure 14 | The *in-vivo* grasping experiment for extracting muscle contraction forces (a)** A standard weight was dropped onto the object to provide externals stimuli. **(b)** The placement of the electromyography (EMG) sensors on the forearm of the human subject.

**Supplementary Table 1** **| The comparisons between the published and the sensor presented in this research with regards to fabrication process, fabrication environment and lead time**

| **Author** | **Fabrication process** | | **Material** | **Lead time** | **High-demanded fabricating environment** | **Reference** |
| --- | --- | --- | --- | --- | --- | --- |
| Boutry et al. | | Photolithography and etching | CNT/Silicone wafer | >1.5 hours | 🗸 | 2 |
| Park et al. | | Solution-based rod casting etc. | GO/PVDF | >24 hours | 🞩 | 15 |
| Makihata et al. | | Wafer bonding, photolithography and etching | Silicon diaphragm | >1 hour | 🗸 | 17 |
| Oh et al. | | Deposition and wet etching | ZnO thin-film transistors | >2 hours | 🗸 | 18 |
| Pyo et al. | | Photolithography and drop casting | NiCr | >0.5 hour | 🗸 | 19 |
| Zhu et al. | | Photolithography and silicon etching | rGO/PDMS | >5 hours | 🗸 | 20 |
| Pang et al. | | Photolithography, etching and molding | Pt/PDMS | >2 hours | 🗸 | 32 |
| Wei et al. (This study) | | 3D Printing | CNT/Graphene /Silicone | 1 hour | 🞩 |  |

**Supplementary Table 2 | The sensitivity of temperature sensing under different pressures**

| **Pressure (KPa)** | **Sensitivity for temperature sensing (°C^-1^)** | |
| --- | --- | --- |
| 0 | 0.27% |  |
| 10  20  30 | 0.25%  0.24%  0.22% |  |
| 40 | 0.19% |  |
| 50 | 0.19% |  |
| 100 | 0.17% |  |
| 150 | 0.17% |  |
| 200 | 0.15% |  |
