# Supplementary material for: Fully 3D printed flexible, conformal and multi-directional tactile sensor with integrated biomimetic and auxetic structure
Source: Commun Eng. 2023 Nov 14;2:80. doi: 10.1038/s44172-023-00131-x (PMC10956052; doi:10.1038/s44172-023-00131-x)
Supplement: Supplementary file 3 — Description of Additional Supplementary File [file 44172_2023_131_MOESM3_ESM.pdf]

Description of Additional Supplementary Files

File name: Supplementary Data 1

Description: The G code for printing the tactile sensor 1

File name: Supplementary Data 2

Description: The G code for printing the tactile sensor 2

File name: Supplementary Data 3

Description: The G code for printing the tactile sensor 3

File name: Supplementary Data 4

Description: The G code for printing the tactile sensor 4

File name: Supplementary Data 5

Description: The python code for the sensorimotor controlling strategy

File name: Supplementary Movie 1

Description: In Supplementary Movie 1, the 3D printing process of the tactile sensor, the finite element simulations for optimizing the structure, and some sensor measurements are presented.
